# The Role of Inflammatory Diet and Vitamin D on the Link between Periodontitis and Cognitive Function: A Mediation Analysis in Older Adults

**DOI:** 10.3390/nu13030924

**Published:** 2021-03-12

**Authors:** João Botelho, Yago Leira, João Viana, Vanessa Machado, Patrícia Lyra, José Manuel Aldrey, Juan Manuel Pías-Peleteiro, Juan Blanco, Tomás Sobrino, José João Mendes

**Affiliations:** 1 Periodontology Department, Instituto Universitário Egas Moniz, 2829-511 Almada, Portugal; vmachado@egasmoniz.edu.pt; 2 Evidence-Based Hub, Clinical Research Unit, Centro de Investigação Interdisciplinar Egas Moniz, 2829-511 Almada, Portugal; jpm.viana.1@gmail.com (J.V.); patricialyra10@gmail.com (P.L.); jmendes@egasmoniz.edu.pt (J.J.M.); 3 Periodontology Unit, UCL Eastman Dental Institute and NIHR UCLH Biomedical Research Centre, University College London, London WC1E 6DE, UK; y.leira@ucl.ac.uk; 4 Periodontology Unit, Faculty of Medicine and Odontology, University of Santiago de Compostela, 15706 Santiago de Compostela, Spain; jblanco@blancoramos.net; 5 Medical-Surgical Dentistry (OMEQUI) Research Group, Health Research Institute of Santiago de Compostela (IDIS), 15706 Santiago de Compostela, Spain; 6 Clinical Neurosciences Research Laboratory, Health Research Institute of Santiago de Compostela (IDIS), 15706 Santiago de Compostela, Spain; tomas.sobrino.moreiras@sergas.es; 7 Dementia Unit, Department of Neurology, Clinical University Hospital, 15706 Santiago de Compostela, Spain; jaldreyv@yahoo.com (J.M.A.); juan.manuel.pias.peleteiro@gmail.com (J.M.P.-P.)

**Keywords:** periodontitis, periodontal disease, inflammation, vitamin D, diet, oral health

## Abstract

Patients suffering from periodontitis are at a higher risk of developing cognitive dysfunction. However, the mediation effect of an inflammatory diet and serum vitamin D levels in this link is unclear. In total, 2062 participants aged 60 years or older with complete periodontal diagnosis and cognitive tests from the National Health and Nutrition Examination Survey (NHANES) 2011–2012 and 2013–2014 were enrolled. The Consortium to Establish a Registry for Alzheimer’s disease (CERAD) word learning subtest (WLT) and CERAD delayed recall test (DRT), the animal fluency test (AFT) and the digit symbol substitution test (DSST) was used. Dietary inflammatory index (DII) was computed via nutrition datasets. Mediation analysis tested the effects of DII and vitamin D levels in the association of mean probing depth (PD) and attachment loss (AL) in all four cognitive tests. Periodontitis patients obtained worse cognitive test scores than periodontally healthy individuals. DII was negatively associated with CERAD-WLT, CERAD-DRT, AFT and DSST, and was estimated to mediate between 9.2% and 36.4% of the total association between periodontitis with cognitive dysfunction (*p* < 0.05). Vitamin D showed a weak association between CERAD-DRT, AFT and DSST and was estimated to between 8.1% and 73.2% of the association between periodontitis and cognitive dysfunction (*p* < 0.05). The association between periodontitis and impaired cognitive function seems to be mediated both by a proinflammatory dietary load and vitamin D deficiency. Future studies should further explore these mediators in the periodontitis-cognitive decline link.

## 1. Introduction

Dementia is a syndrome of progressive memory loss and impaired cognitive ability, impacting everyday activities [1]. This condition is estimated to affect 131.5 million people worldwide by 2050, with severe impairment on patients’ quality of life [2]. Currently, the main focus of dementia research relies on its neurological characteristics, mechanisms and novel therapeutic breakthroughs [3]. To date, understanding dementia and its risk factors has been greatly accomplished through large population-based surveys [3,4], whose results have expanded knowledge on new oral health-related risk factors, such as tooth loss [5,6,7] and periodontitis [8,9,10,11,12,13].

Periodontitis is an irreversible disease that can lead to tooth loss [14,15]. This non-communicable disease is characterized by a polymicrobial dysbiotic infection of the periodontium [16,17]. In cases of unresolved periodontitis, teeth may be lost entirely and deteriorate quality of life in a reversible manner if treatment is delivered [18]. Despite the association between periodontitis and dementia being far from fully comprehended [19], demented patients tend to have worse periodontal status [8,13,20], and systemic inflammation may mediate the association between periodontitis and cognitive impairment [8].

Likewise, evidence shows an apparent role of diet in both dementia and periodontitis. Poor diet and vitamin B deficits are associated with cognitive decline [21,22]. In fact, adherence to proinflammatory dietary patterns was associated with greater cognitive decline [23,24], while dietary anti-inflammatory patterns reduce the inflammatory burden in periodontitis lesions [25]. Additionally, vitamin D deficiency has been growing attention reasonably in dementia [26,27,28] and periodontitis [29] since lower vitamin D intake associates with a higher risk of both conditions. However, the mediating role of inflammatory diet and vitamin D on the association of periodontal status and cognitive function has never been investigated.

Therefore, we aimed to explore the association of periodontitis with cognitive functioning and the mediation effect of an inflammatory diet and serum vitamin D levels in this link.

## 2. Materials and Methods

### 2.1. Study Design and Participants

A secondary analysis was carried out on two datasets from the National Health and Nutrition Examination Survey (NHANES). NHANES is a stratified multistage national representative survey with a civilian noninstitutionalized population in fifty states of the USA and the District of Columbia. All details on sampling, design, medical records and periodontal data collections can be found at www.cdc.gov/nchs/nhanes.htm (accessed on 30 August 2020). Both NHANES 2011–2012 and 2013–2014 datasets were reviewed and approved by the Centers for Disease Control (CDC) and Prevention National Increase for Health Statistics Research (NCHS) Ethics Review Board, and all included participants provided written informed consent. In the NHANES 2011–2014, a subsample of the population aged 60 years and above were asked to carry out cognitive tests. The survey also included a periodontal examination in all dentition. For the purpose of this study, we have included those participants that completed both periodontal examination and cognitive function tests.

This study followed the strengthening of the reporting of observational studies in epidemiology (STROBE) guidelines [30] (Supplementary Table S1).

### 2.2. Cognitive Assessment

Cognitive function was measured using a series of assessments [31], including (1) word learning and recall modules from the Consortium to Establish a Registry for Alzheimer’s disease (CERAD) [32]; (2) the animal fluency test [33]; and (3) the Digit Symbol Substitution test (DSST) [34].

The CERAD word learning subtest (CERAD-WLT) assesses immediate and delayed learning ability for new verbal information (memory sub-domain) [32]. The test consists of three consecutive learning trials and a delayed recall. For the learning trials, participants are instructed to read aloud 10 unrelated words, one at a time, as they are presented. Immediately following the presentation of the words, participants recall as many words as possible. In each of the three learning trials, the order of the 10 words is changed. The maximum score possible on each trial is 10. In NHANES, the words for the learning trials were presented in large, bolded letters on a computer monitor. Participants, who were unable to read due to literacy or a visual impairment, were asked to repeat each word after it was read by the interviewer. The delayed word recall (CERAD delayed recall test (CREAD-DRT)) occurred after the other two cognitive exercises (animal fluency and DSST) were completed (approximately 8–10 min from the start of the word learning trials).

The animal fluency test examines categorical verbal fluency, a component of executive function [33]. Participants are asked to name as many animals as possible in one minute. A point is given for each named animal. In NHANES, participants first were asked to name three items of clothing, another verbal fluency category, as a practice test. Participants who could not name three articles of clothing did not continue with the animal fluency exercise.

The digit symbol substitution test (DSST) relies on processing speed, sustained attention, and working memory [34]. The exercise is conducted using a paper form that has a key at the top containing 9 numbers paired with symbols. Participants have 2 min to copy the corresponding symbols in the 133 boxes that adjoin the numbers. The score is the total number of correct matches. A sample practice test is administered before the participants begin the main test. In NHANES, participants who could not correctly match the symbols with the numbers on their own during the pretest practice did not continue.

### 2.3. Periodontal Assessment

A full-mouth periodontal examination was conducted by calibrated examiners as described elsewhere [35]. Pocket depth (PD) and attachment loss (AL) were recorded at six sites per tooth (mesio-buccal, mid-buccal, disto-buccal, mesio-lingual (or palatal), mid-lingual (or palatal), and disto-lingual (or palatal)). Periodontitis was defined as the presence of a minimum of 2 or more sites with AL  ≥ 3 mm and PD ≥ 4 mm or one site with PD ≥ 5 mm [36]. The number of missing teeth was also recorded.

### 2.4. Dietary Inflammatory Index (DII)

The DII is a literature-based instrument [37] that computes the inflammatory properties of a diet, based on the association of certain food and dietary constituents on defined inflammatory hallmarks: CRP, TNF-α and IL-1β, IL-4, IL-6 and IL-10. From a possible total of 45 food parameters, the final score is a continuous measure, interpreted as strongly anti-inflammatory (the lowest score) to strongly proinflammatory (the highest score), respectively [37].

In this study, DII was computed using the NHANES database for a dietary interview for the total nutrient intakes. We calculated the DII score for the 26 food parameters available. Eugenol, garlic, ginger, niacin, onion, saffron, saturated fat, trans fat, turmeric, green/black tea, flavan-3-ol, flavones, flavonols, flavanones, anthocyanidins, isoflavones, pepper, thyme/oregano, rosemary were not included because no information was available. First, we calculated the z-score of each food parameter and for each participant. Second, each individual z-score was converted to a centered percentile. Third, each centered percentile was multiplied by the standardized overall inflammatory effect score [37]. Finally, the DII score was summed and obtained for each participant.

### 2.5. Sociodemographic and Health-Related Variables

In the sociodemographic variables, we included age, gender, ethnicity, educational level, marital status and family income to poverty ratio. Current smokers were those who had smoked ≥100 cigarettes during their lifetime and were still active smokers. Former smokers were defined as those who had smoked ≥100 cigarettes during their lifetime but had stopped. Those who had smoked <100 cigarettes during their lifetimes were categorized as never smokers. Minutes of sedentarism was used as a proxy of physical activity.

To assess participant’s systemic status, we considered the number of self-reported medical conditions as an aggregate continuous variable to note the presence of asthma, congestive heart failure, coronary heart disease, angina, stroke, heart attack, emphysema, overweight, bronchitis, liver conditions, thyroid conditions and cancer. Considering the strong association of hypertension [38] and diabetes [39,40] with periodontitis in the NHANES, we included these variables separately. The presence of hypertension was defined according to the presence of average systolic blood pressure > 140 mmHg or average diastolic blood pressure > 90 mmHg [41]. The presence of diabetes was recorded as a self-reported measure from a previous report of a doctor or a health professional. Body mass index (BMI) was calculated using the standard formula weight/height [2,42].

### 2.6. Biochemical Parameters

Data from lipid fractions (including HDL cholesterol (mg/dL), LDL cholesterol (mg/dL), total cholesterol (mg/dL), and triglycerides (mg/dL)) were analyzed from blood specimens [43,44]. WBC count (10^9^/L) were analyses through volume, conductivity and scatter technology [43,44]. In addition, serum levels of total vitamin D (nmol/L) [43,44] were calculated by the sum of 25-hydroxyvitamin D2 and 25-hydroxyvitamin D3.

### 2.7. Statistical Analysis

Datasets from the NHANES 2011–2012 and 2013–2014 were handled through SPSS version 25.0 for Macintosh (Armonk, NY, USA, IBM Corp.). Concerning the periodontal diagnosis, data were exported to a specific MO Excel with appropriate algorithms that were derived from formulating the periodontal case definition [45,46]. We report continuous and categorical variables through mean ± standard deviation (SD) and a number of cases (n) with percentage (%), respectively. All statistical analyses were computed in R. After inspection of data normality and homoscedasticity; we employed the t-test to compare mean values according to the periodontal status. Additionally, the chi-squared test was used for the comparison of categorical variables.

**Figure 1:**
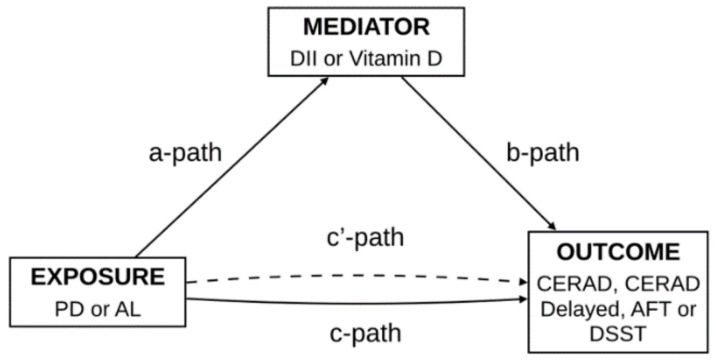
Path diagram of the mediation analysis models.

Finally, a mediation analysis was carried out to examine the mediating effect of DII and vitamin D levels in the association of two periodontal measures (mean PD and Mean AL) with each cognitive functioning test (CERAD-WLT, CERAD-DRT, animal fluency and DSST) (Figure 1). The mediating effect of DII and vitamin D in the association of PD and CAL with all four cognitive function tests were carried out using the r package “lavaan”. Mediation analysis was done through the establishment of three pathways (a, b and c) (Figure 1). For this purpose, we set three pathways: (1) exposure to mediator; (2) mediator to outcome (direct effect); and (3) exposure to outcome (total effect). The total effect reflected the sum of a direct effect and a mediated (indirect) effect. The percentage of the mediated effect was calculated using the following formula: (mediated effect/total effect) × 100 [47]. Bootstrapping was used for significance testing for mediation analysis.

A significance level of 5% was set in all inferential analyses.

## 3. Results

### 3.1. General Characteristics

**Figure 2:**
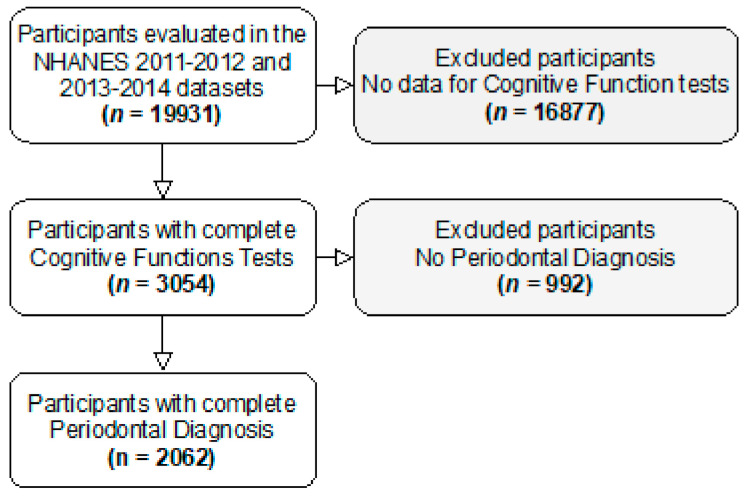
Participants flowchart.

From 3054 participants that have completed cognitive function tests, 2062 had complete periodontal data and were included in our analyses (Figure 2).

Table 1 presents general characteristics of study participants weighted to the US population according to the presence/absence of periodontitis.

Statistically significant differences were observed between those with and without periodontitis in terms of sociodemographic data (i.e., gender, race, education level, marital status and family income/poverty ratio) (all *p* < 0.001).

Compared to non-periodontitis individuals, participants diagnosed with periodontitis were more frequently current smokers (14.4% vs. 5.0%, *p* < 0.001), had more often a previous history of hypertension (30.9% vs. 25.5%, *p* = 0.014) and diabetes (26.7% vs. 15.8%, *p* = 0.001), and were less physically active (minutes of sedentarism: 462.2 vs. 435.4 min, *p* < 0.001).

As expected, clinical parameters of periodontal inflammation and destruction were increased in the periodontal group (PD: 1.87 vs. 1.13 mm, *p* < 0.001 and AL: 2.51 vs. 1.34 mm, *p* < 0.001).

Cognitive performance of periodontitis subjects was significantly worse than periodontally healthy participants (CERAD-WLT: 17.2 vs. 19.2, *p* < 0.001; CERAD-DRT: 5.4 vs. 6.2, *p* < 0.001; animal fluency test: 15.0 vs. 17.1, *p* < 0.001; DSST: 40.9 vs. 50.2, *p* < 0.001).

A more proinflammatory diet was observed in periodontitis subjects (DII: −0.05 vs. −0.32, *p* < 0.001). Likewise, the biochemical analysis showed that peripheral levels of vitamin D and HDL were decreased, while WBC counts were elevated in the periodontitis group compared to those without periodontitis (all *p* < 0.001).

### 3.2. Mediation Analysis

#### 3.2.1. Proinflammatory Diet

There was evidence that the association between periodontitis and poor performance in the four cognitive tests was mediated by a proinflammatory diet.

Mean PD and AL were negatively associated with animal fluency test and DSST and positively associated with DII (Table 2, Table 3, Table 4 and Table 5). Further, DII was negatively associated with CERAD-WLT, CERAD-DRT, animal fluency test and DSST (Table 2, Table 3, Table 4 and Table 5). It was estimated that between 9.2% and 36.4% of the total association between periodontitis with cognitive dysfunction was mediated by DII (*p* < 0.05).

#### 3.2.2. Vitamin D Deficit

Similarly, linear regression analyses showed that reduced levels of vitamin D also mediated the association between periodontitis and diminished cognitive function.

A negative association was found between mean AL and animal fluency test, mean PD and AL with DSST (Table 4 and Table 5). Further, both mean PD and AL were negatively associated with circulating vitamin D levels. In addition, a weak association was observed between vitamin D and CERAD-DRT, animal fluency test and DSST (Table 3, Table 4 and Table 5). The mediation effect of vitamin D levels in the total association between periodontitis and cognitive function was estimated to range between 8.1% and 73.2% (*p* < 0.05).

## 4. Discussion

In the present study, community-dwelling US older adults diagnosed with periodontitis exhibited worse cognitive function than periodontally healthy individuals. In the adjusted mediation analysis, a more inflammatory dietary load and lower vitamin D levels showed to significantly mediate the association of periodontitis with poor performance in all cognitive tests.

The inflammatory dietary burden was quantified through the DII, a comprehensive and literature-based tool [37,48] previously linked with the variation of inflammatory surrogates [49,50,51] and implicated in systemic diseases [52,53]. In all, DII remarkably mediated the association of periodontitis and cognitive decline, which may be seen as novel. The role of an overly inflammatory diet in dementia has been consistently proposed [21,54,55], as it may accelerate its progression through the trigger of neuroinflammation pathways. As a result, an individual with an inflammatory imbalance due to diet or inflammatory conditions (i.e., periodontitis) may precipitate biological mechanisms that may worsen cognitive decline. Thus, resolving both factors through proper diet and periodontal treatment might have the potential to mitigate this neuroinflammatory processes, though at this stage this is merely speculative.

Additionally, the association of low circulating levels of vitamin D with periodontitis has been well studied [29,56]. The same was found for dementia, where individuals with vitamin D deficiency have a higher risk of cognitive impairment and dementia [57,58]. In fact, vitamin D regulates calcium balance, Aβ deposition and has antioxidant and anti-inflammatory properties in Alzheimer’s disease [55,59].

Overall, this study presents methodological strengths and limitations that deserve consideration. The periodontal diagnosis was based on a full-mouth examination of six sites per tooth, which is considered the gold-standard approach, with low bias risk [45,60,61]. This contrasts with a previous study where the partial-mouth inspection was carried out [8]. Furthermore, cognitive function was assessed through the application of four tests, which enlightens the perception of the individual cognitive status. Further, the presence of diabetes and hypertension was confirmed through recognized clinical standards, despite the presence of the remaining pathologies that were based on self-reports. On the other hand, the cross-sectional design of the NHANES limits any extrapolation of causality or temporal association. The NHANES 2011–2012 and 2013–2014 lack gingival bleeding data, precluding a more exhaustive analysis on periodontal inflammation and the computation of the periodontal inflamed surface area that was previously linked in the association of periodontitis and cognitive functioning [8]. In addition, the DII score was the result of 26 out of 45 possible food parameters and, therefore, this could contribute to the underestimation of these results, yet this approach has been previously employed [62,63]. Also, the food questionnaire concerns a self-report from the past 24 h span, and this may be seen as a limitation [51]. Long-term prospective studies and well-designed interventional trials are warranted to enlighten the association between periodontitis and cognitive decline and how the inflammatory dietary burden and vitamin D levels can exert such a mediation effect.

## 5. Conclusions

Periodontitis was associated with significantly worse cognitive performance, and periodontitis patients reported a proinflammatory prone diet. Furthermore, serum vitamin D levels were decreased in periodontitis patients. Ultimately, the link between periodontitis and an impaired cognitive function seems to be mediated both by a proinflammatory dietary load and vitamin D deficits. Future studies should further explore the periodontitis-cognitive decline link, as well as the mechanisms through which the inflammatory dietary burden and low vitamin D levels are involved in this association.

## Figures and Tables

**Table 1:** Sample characteristics according to periodontal status (*n* = 2062).

	No Periodontitis (*n* = 625)	Periodontitis (*n* = 1437)	*p*-Value
Age (years), mean (SD)	68.74 (0.27)	69.0 (0.18)	0.395
Gender, *n* (%)			
Males	228 (36.5)	816 (56.8)	<0.001
Females	397 (63.5)	621 (43.2)	
Race/ethnicity, *n* (%)			
Mexican American	50 (8.0)	162 (11.2)	<0.001
Non-Hispanic White	46 (7.3)	173 (12.0)	
Non-Hispanic Black	373 (59.7)	559 (38.9)	
Other Hispanic	92 (14.7)	371 (25.8)	
Other race	64 (10.2)	172 (12)	
Education level, *n* (%)			
<High school	39 (6.2)	215 (14.9)	<0.001
High school	179 (28.6)	523 (36.3)	
>High school	407 (65.1)	699 (48.6)	
Smoking status, *n* (%)			
Never	390 (62.4)	687 (48.5)	
Former	204 (32.6)	530 (36.9)	
Current	31 (5.0)	209 (14.6)	
BMI (kg/m^2^), mean (SD)	28.8 (0.24)	28.7 (0.16)	0.408
Family income/poverty ratio, mean (SD)	2.98 (1.69)	2.40 (1.62)	<0.001
Marital status			
Single	39 (6.2)	94 (6.5)	0.012
Married/living with a partner	404 (64.6)	831 (57.8)	
Divorced/separated/widowed	182 (29.1)	512 (35.6)	
Chronic medical conditions, mean (SD)	0.73 (0.44)	0.75 (0.43)	0.351
Diabetes, *n* (%)	99 (15.8)	340 (26.7)	0.001
Hypertension, *n* (%)	159 (25.5)	439 (30.9)	0.014
Minutes of sedentarism (min), mean (SD)	435.4 (577.4)	462.2 (895.7)	<0.001
Cognitive function, mean (SD)			
CERAD word learning	19.2 (6.1)	17.2 (6.8)	<0.001
CERAD delayed recall	6.2 (2.6)	5.4 (2.7)	<0.001
Animal fluency test	17.1 (6.7)	15.0 (6.9)	<0.001
DSST	50.2 (20.4)	40.9 (21.1)	<0.001
Periodontal measurements, mean (SD)			
PD (mm)	1.13 (0.32)	1.87 (0.80)	<0.001
AL (mm)	1.34 (0.42)	2.51 (1.23)	<0.001
Missing teeth	6.9 (7.3)	9.1 (7.2)	<0.001
DII, mean (SD)	−0.32 (1.81)	−0.05 (1.80)	<0.001
Biochemical parameters, mean (SD)			
Vitamin D (nmol/L)	82.92 (30.47)	72.87 (30.67)	<0.001
WBC (10^9^/L)	6.57 (1.79)	7.02 (2.56)	<0.001
HDL (mg/dL)	57.57 (16.29)	55.20 (16.22)	<0.001
LDL (mg/dL)	112.00 (37.27)	110.07 (36.49)	0.438
Total cholesterol (mg/dL)	193.59 (42.49)	190.68 (41.06)	0.065
Triglycerides (mg/dL)	118.07 (63.25)	121.25 (73.92)	0.505

Abbreviations: BMI, body mass index; CERAD, Consortium to Establish a Registry for Alzheimer’s disease; DSST, digit symbol substitution test; AL, attachment loss; PD, pocket depth; WBC, white blood cells; DII, dietary inflammatory index; HDL: high-density lipoprotein; LDL: low-density lipoprotein.

**Table 2:** Estimates (SE) of mediation analysis for the association between periodontitis and CERAD word learning test.

Exposure: PD and Outcome: CERAD Word Learning Test						
Mediator	Exposure to Mediator	Mediator to Outcome	Direct Effect	Mediated (Indirect) Effect	Total Effect (Exposure to Outcome)	Proportion Mediated (%)
DII	0.21 (0.08) **	−0.37 (0.11) ***	−0.41 (0.25)	−0.08 (0.04) *	−0.48 (0.25)	16.2
Vitamin D (nmol/L)	−6.24 (1.29) ***	0.01 (0.01)	−0.36 (0.27)	−0.06 (0.04)	−0.42 (0.27)	-
**Exposure: AL and Outcome: CERAD Word Learning Test**						
**Mediator**	**Exposure to Mediator**	**Mediator to Outcome**	**Direct Effect**	**Mediated (Indirect) Effect**	**Total Effect (Exposure to Outcome)**	**Proportion** **Mediated (%)**
DII	0.14 (0.05) **	−0.37 (0.11) ***	−0.20 (0.17)	−0.05 (0.02) *	−0.26 (0.17)	19.9
Vitamin D (nmol/L)	−3.80 (0.84) ***	0.05 (0.03)	−0.24 (0.18)	−0.04 (0.03)	−0.28 (0.18)	-

All models adjusted for sociodemographic variables (age, gender, race, education, marital status, family income/poverty ratio), health behaviors (minutes of sedentarism and smoking habit), body mass index, missing teeth and systemic status (number of chronic medical conditions, hypertension, diabetes, HDL cholesterol, LDL cholesterol, total cholesterol, triglycerides). * *p* < 0.05; ** *p* < 0.01; *** *p* < 0.001. Abbreviations: AL, attachment loss; PD, pocket depth; DII, dietary inflammatory index; CERAD, Consortium to Establish a Registry for Alzheimer’s disease.

**Table 3:** Estimates (SE) of mediation analysis for the association between periodontitis and CERAD Delayed Recall Test.

Exposure: PD and Outcome: CERAD Delayed Recall Test						
Mediator	Exposure to Mediator	Mediator to Outcome	Direct Effect	Mediated (Indirect) Effect	Total Effect (Exposure to Outcome)	Proportion Mediated (%)
DII	0.21 (0.08) **	−0.13 (0.05) ***	−0.10 (0.11)	−0.03 (0.01) *	−0.12 (0.11)	22.8
Vitamin D (nmol/L)	−6.24 (1.29) ***	0.01 (0.00) *	−0.06 (0.11)	−0.05 (0.02) *	−0.11 (0.11)	73.2
**Exposure: AL and Outcome: Delayed Recall Test**						
**Mediator**	**Exposure to Mediator**	**Mediator to Outcome**	**Direct Effect**	**Mediated (Indirect) Effect**	**Total Effect (Exposure to Outcome)**	**Proportion** **Mediated (%)**
DII	0.14 (0.05) **	−0.13 (0.05) **	−0.03 (0.07)	−0.02 (0.01) *	−0.05 (0.07)	36.4
Vitamin D (nmol/L)	−3.80 (0.84) ***	0.01 (0.00) *	−0.04 (0.07)	−0.03 (0.01) *	−0.06 (0.07)	63.3

All models adjusted for sociodemographic variables (age, gender, race, education, marital status, family income/poverty ratio), health behaviors (minutes of sedentarism and smoking habit), body mass index, missing teeth and systemic status (number of chronic medical conditions, hypertension, diabetes, HDL cholesterol, LDL cholesterol, total cholesterol, triglycerides). * *p* < 0.05; ** *p* < 0.01; *** *p* < 0.001. Abbreviations: AL, attachment loss; PD, pocket depth; DII, dietary inflammatory index; CERAD, Consortium to Establish a Registry for Alzheimer’s disease.

**Table 4:** Estimates (SE) of mediation analysis for the association between periodontitis and animal fluency test.

Exposure: PD and Outcome: Animal Fluency Test						
Mediator	Exposure to Mediator	Mediator to Outcome	Direct Effect	Mediated (Indirect) Effect	Total Effect (Exposure to Outcome)	Proportion Mediated (%)
DII	0.21 (0.08) **	−0.46 (0.11) ***	−0.49 (0.27)	−0.10 (0.05) *	−0.58 (0.27) *	16.7
Vitamin D (nmol/L)	−6.24 (1.29) ***	0.01 (0.01) *	−0.42 (0.28)	−0.08 (0.05) *	−0.51 (0.28)	12.2
**Exposure** **: AL and Outcome: Animal Fluency Test**						
**Mediator**	**Exposure to Mediator**	**Mediator to Outcome**	**Direct Effect**	**Mediated (Indirect) Effect**	**Total Effect (Exposure to Outcome)**	**Proportion** **Mediated (%)**
DII	0.14 (0.05) **	−0.46 (0.11) ***	−0.32 (0.18)	−0.07 (0.03) *	−0.39 (0.18) *	16.5
Vitamin D (nmol/L)	−3.80 (0.84) ***	0.01 (0.01)	−0.34 (0.18)	−0.05 (0.03)	−0.39 (0.18) *	-

All models adjusted for sociodemographic variables (age, gender, race, education, marital status, family income/poverty ratio), health behaviors (minutes of sedentarism and smoking habit), body mass index, missing teeth and systemic status (number of chronic medical conditions, hypertension, diabetes, HDL cholesterol, LDL cholesterol, total cholesterol, triglycerides). * *p* < 0.05; ** *p* < 0.01; *** *p* < 0.001. Abbreviations: AL, attachment loss; PD, pocket depth; DII, dietary inflammatory index.

**Table 5:** Estimates (SE) of mediation analysis for the association between periodontitis and DSST.

Exposure: PD and Outcome: DSST						
Mediator	Exposure to Mediator	Mediator to Outcome	Direct Effect	Mediated (Indirect) Effect	Total Effect (Exposure to Outcome)	Proportion Mediated (%)
DII	0.21 (0.08) **	−1.70 (0.30) ***	−3.23 (0.72) ***	−0.36 (0.16) *	−3.24 (0.72) ***	11.0
Vitamin D (nmol/L)	−6.24 (1.29) ***	0.06 (0.02) ***	−3.64 (0.76) ***	−0.40 (0.15) *	−4.03 (0.76) ***	9.3
**Exposure** **: AL and Outcome: DSST**						
**Mediator**	**Exposure to Mediator**	**Mediator to Outcome**	**Direct Effect**	**Mediated (Indirect) Effect**	**Total Effect (Exposure to Outcome)**	**Proportion Mediated (%)**
DII	0.14 (0.05) **	−1.69 (0.30) ***	−2.33 (0.48) ***	−0.24 (0.10) *	−2.57 (0.48) ***	9.2
Vitamin D (nmol/L)	−3.80 (0.84) ***	0.06 (0.02) ***	−2.56 (0.50) ***	−0.24 (0.09) *	−2.80 (0.49) ***	8.1

All models adjusted for sociodemographic variables (age, gender, race, education, marital status, family income/poverty ratio), health behaviors (minutes of sedentarism and smoking habit), body mass index, missing teeth and systemic status (number of chronic medical conditions, hypertension, diabetes, HDL cholesterol, LDL cholesterol, total cholesterol, triglycerides). * *p* < 0.05; ** *p* < 0.01; *** *p* < 0.001. Abbreviations: AL, attachment loss; PD, pocket depth; DII, dietary inflammatory index; DSST, Digit Symbol Substitution Test.

## Data Availability

Data are available at the NHANES website.

## Supplementary Materials

The following are available online at https://www.mdpi.com/2072-6643/13/3/924/s1, Table S1: STROBE checklist.
